# The effects of cognitive-motor dual-task training on athletes’ cognition and motor performance

**DOI:** 10.3389/fpsyg.2024.1284787

**Published:** 2024-02-08

**Authors:** Junyu Wu, Peng Qiu, Shuaibing Lv, Mingxian Chen, Youqiang Li

**Affiliations:** ^1^School of Physical Education, Shanghai University of Sport, Shanghai, China; ^2^Department of Rehabilitation Medicine, The First Affiliated Hospital of Wenzhou Medical University, Wenzhou, Zhejiang, China; ^3^School of Exercise and Health, Shanghai University of Sport, Shanghai, China

**Keywords:** dual-task, cognitive-motor dual-task, athletes, cognitive performance, motor performance

## Abstract

**Background:**

Cognitive-Motor Dual Task (CMDT) training has been widely utilized in rehabilitation and sports practice. However, whether CMDT training can better enhance athletes’ cognitive-motor performance compared to traditional single-task (ST) training remains unclear.

**Method:**

A systematic review that complied with PRISMA was carried out (Prospero registration number: CRD42023443594). The electronic databases used for the systematic literature search from the beginning through 13 June 2023, included Web of Science, Embase, PubMed, and the Cochrane Library. After obtaining the initial literature, two researchers independently assessed it based on inclusion and exclusion criteria. Finally, the included literature was analyzed to compare the differences between ST training and CMDT training.

**Results:**

After screening 2,094 articles, we included 10 acute studies and 7 chronic studies.

**Conclusion:**

This systematic review shows that athletes typically show a degradation of performance in CMDT situations as opposed to ST when evaluated transversally. However, this performance decline is notably reduced following longitudinal training in CMDT, indicating the effectiveness of sustained CMDT training in enhancing cognitive-motor performance under dual-task conditions. Our study provides new insights into the application of CMDT in the field of sports training. Practitioners can utilize CMDT to assess athletic skill levels or optimize cognitive-motor performance of athletes, taking into account the specific needs of each sport.

**Systematic review registration:**

https://www.crd.york.ac.uk/prospero, identifier CRD42023443594.

## 1 Introduction

In the sphere of athletic development, it is argued that a training regimen which mirrors, to the highest degree possible, the demands inherent to actual competition yields the most substantial transfer effects on athletes’ competitive performance (Murphy et al., 2016). Consequently, optimal training is posited to be that which converges with the reality of competition (Halouani et al., 2014; Murphy et al., 2016). The rapid advancement of modern competitive sports, along with the corresponding increase in competitive intensity among athletes, has given rise to this concept. Superior performances are often the emergent properties of a multifaceted matrix that intricately intertwines components such as rigorous training (Laursen and Jenkins, 2002; Smith, 2003; Sarmento et al., 2018), honed skills (Hrysomallis, 2011; Suchomel et al., 2016), and inherent talents (Smith, 2003; Breitbach et al., 2014; Varillas-Delgado et al., 2022). The progressive strides made in the fields of sports science and sports psychology have incrementally augmented our understanding of competition-centric training. Historically, the focus of inquiry gravitated predominantly toward the tangible, physical aspects of training, which included elements like fitness enhancement and technical skill refinement (Beattie et al., 2014; Wortman et al., 2021). However, the present-day narrative has witnessed a paradigmatic shift, with a surge in the number of researchers turning their investigative lens toward the pivotal role cognition plays within the sphere of athletic training (Broadbent et al., 2015; Slimani et al., 2016; Bühlmayer et al., 2017; Emirzeoğlu and Ülger, 2021). In the crucible of real-world competition, athletes are mandated to draw from a well-rounded skill set (Broadbent et al., 2015). This necessitates not only a sturdy foundation of physical robustness and technical prowess but also the ability to swiftly seize evanescent opportunities amidst complex athletic environments (Fuster et al., 2021). This dexterity enables athletes to execute a variety of technical maneuvers in a timely fashion, thereby optimizing their victory potential (Sabarit et al., 2020).

Consider the paradigm of a basketball match. A point guard, tasked with both dribbling and scanning the court, must maintain a keen awareness of the positions of teammates and opponents. This situational awareness allows the point guard to distribute the ball optimally, entrusting it to the player with the greatest opportunity at a given moment, hence setting the stage for an offensive maneuver. This scenario exemplifies the characteristic features of dual-tasking (DT; Bronstein et al., 2019), a subject of growing interest in contemporary sports research. Furthermore, extending this concept to incorporate the notion of “incorporated/added DT” as proposed by Herold et al. (2018) provides a more nuanced understanding of DT in sports contexts. This approach, differentiating from the traditional DT framework, involves the intentional addition of an extra cognitive task alongside the primary motor activity. For instance, a point guard engaged in regular dribbling and court scanning might also be tasked with an additional memory or attention challenge. This integrated approach enables a more precise evaluation of the interplay and coordination between cognitive and motor tasks, offering a means to control and quantify cognitive load in real-time sports situations. The application of “incorporated/added DT” methodology not only mirrors the complex realities of sports competitions but also allows for a deeper exploration into how athletes maintain a balance between motor skills and situational awareness under varying cognitive demands. Insights gained from this perspective are crucial for developing training methods that enhance cognitive-motor coordination and overall athletic performance, particularly in sports that demand high levels of strategic thinking and quick decision-making.

Traditional athletic training acknowledges the importance of periodized arrangement of individual training tasks, such as technique, physical fitness, tactics, and psychology, for optimizing athletes’ performance to the maximum extent (Issurin, 2010; Hartmann et al., 2015). However, a fundamental difference exists between the actual demands faced by athletes who complete cognitive and motor tasks simultaneously in competitive scenarios and the training mode that involves sequentially completing technical and tactical exercises. This discrepancy may limit the transference effect of training. Therefore, researchers in sports science and psychology have gradually begun to pay attention to the cognitive-motor dual task (CMDT) training (Gabbett et al., 2011), which creatively combines specialized athletic techniques with cognitive tasks in the hopes of enhancing athletes’ performance in actual competitions.

In the field of Cognitive-Motor Dual-Task (CMDT) training, distinct streams of research have emerged, each focusing on different applications and outcomes. Athletic training research primarily seeks theoretical and methodological advancement for performance enhancement. In this domain, studies have explored how CMDT can be utilized for the simultaneous development of physical and cognitive skills in professional athletes, such as in the training routines of NBA players like Jeremy Lin, who performs dribbling and arithmetic tasks concurrently. On the other hand, athletic rehabilitation research has been more focused on using CMDT for post-injury recovery. Much of the current evidence for the benefits of CMDT training, surprisingly, did not originate from athletic training research but rather from the fields of athletic rehabilitation and athletic practice (Pang et al., 2018; Gallou-Guyot et al., 2020; Tuena et al., 2023). CMDT has shown promise in improving patients’ neuro-muscular functions and motor-cognitive abilities, aiding in the recovery of normal functions post-injury. This is evident in the improvement of physical functions and cognitive-motor performance in individuals with conditions like Parkinson’s disease (Pereira-Pedro et al., 2022), stroke (Liu et al., 2017; Zhou et al., 2021), falls (Lord and Close, 2018). Furthermore, clinical research has demonstrated the significant contributions of CMDT in clinical risk assessment and prognostic evaluation. For instance, CMDT approaches combining walking and cognitive tasks are used to assess concussion risks in athletes or evaluate recovery statuses in concussion patients (Howell et al., 2017a,b).

Despite the accumulation of substantial evidence supporting CMDT training in areas such as rehabilitation therapy, current studies on CMDT within the sports science community is still in its infancy (Moreira et al., 2021). The exploration of CMDT training in the field of sports training remains limited, and the mechanisms and temporal progression of CMDT adaptation are still not fully understood (Moreira et al., 2021). For instance, while existing evidence has affirmed the potential benefits of CMDT on motor-cognitive performance, some studies have pointed out that the execution of DT in open-skilled sports is subject to strict time constraints (Baumeister, 1984). This may lead to an excessive cognitive load on individuals in a short time, causing a drastic decline in overall performance. Moreover, although previous systematic reviews have discussed the impact of DT training on athletes, the literature includes an excess of single cognitive type DT (Moreira et al., 2021), which clearly does not align with task characteristics during sports competition. Finally, due to considerable heterogeneity in CMDT intervention strategies for athletes in different sports, the applicability of this method in the field of sports remains indeterminable. Thus, the objective of this article is to systematically evaluate the impact of CMDT on the cognitive functions and athletic performance of athletes, in the hopes of providing a theoretical foundation for subsequent research, and offering guidance for coaches and related practitioners in formulating and adjusting sports training plans.

## 2 Methods

This systematic review is in alignment with the standards set by the “Preferred Reporting Items for Systematic Reviews and Meta-Analyses” (PRISMA; Moher et al., 2009; Prospero registration number: CRD42023443594), and the included literature is organized and analyzed in accordance with its requirements. Given the observed considerable heterogeneity in the methodologies and measurement methods of the included studies (please refer to the Supplementary Figures S1–S6), we are unable to conduct a meta-analysis.

### 2.1 Search strategy

This systematic review encompasses all literature available up to June 2023. Researchers Junyu Wu and Peng Qiu independently searched the PubMed, Web of Science (WOS), Embase, and Cochrane Library databases to find studies relevant to the topic. The search strategy was developed based on previous systematic reviews and was improved upon (Moreira et al., 2021). It is divided into the following parts: (1) dual task and its synonyms, (2) athletes and their synonyms, (3) athletic performance and its synonyms, (4) cognitive performance and its synonyms. Apart from the third and fourth components, which are joined by “OR,” the rest of the parts are interconnected by “AND,” constituting the search equation. The specific search string is as follows: “Cognitive motor” OR “dual task paradigm” OR “dual-task” OR “dual task” OR “double task” OR “multi-task” OR “divided attention” OR “secondary task” OR “second task” AND “athletes” OR “players” OR “player” OR “athlete” AND “working memory” OR “visual” OR “decision making” OR “gaze behavior” OR “attention” OR “athletic Performance” OR “athletic performances” OR “sports performance” OR “performance, sports.”

### 2.2 Criteria for inclusion and exclusion

This systematic review adopts the PICO principles, as espoused by the Cochrane Collaboration, to establish the criteria for document inclusion. The established criteria are as follows: (1) Participants in the study comprise athletes at any competency level, emphasizing the universality of Cognitive-Motor Dual Tasking (CMDT). (2) The study concurrently reports on athletes’ performance under both single-task (ST) and dual-task (DT) environments. (3) At a minimum, either cognitive performance or athletic performance of the athletes is reported. Exclusion criteria dictate the removal of a document under any of the following circumstances: (1) The presence of biomechanical studies investigating conditions under both ST and DT. (2) The participants are injured, cognitively impaired, or physically handicapped. (3) Dual-tasking does not involve a motor task or a cognitive task but merely constitutes the pairing of two tasks of the same type.

### 2.3 Data extraction

Data were extracted based on the established inclusion criteria, with the final data comprising the following elements: (1) Fundamental bibliographic details, including author names, title, and the year of publication; (2) Sample size; (3) Characteristics of the participants, including age, gender, training history, and level of skill; (4) Types of intervention strategies, encompassing acute or training interventions, duration of intervention periods, frequency, volume of training, specific intervention methods, etc.; and (5) Outcome measures, including primary outcome indicators and associated results. In cases of missing data within the literature, we reached out to the authors through email to request the missing data. We used Web Plot Digitizer software (Version 4.0; E, United States) to extract result data (mean ± standard deviation) reported only in graphic form. Two researchers independently extracted the data using tables, then merged the data. In cases of disagreement, a third researcher was consulted for a final decision.

Long-term studies are defined as those in which the intervention plan and period are clearly reported, with ST serving as the control group and CMDT as the experimental group. If there was no apparent CMDT plan and period, or if only a one-time report of ST and CMDT performances was provided, it was classified as an acute study. More accurately, acute studies only conduct transversal ST/CMDT evaluation, not training. In the incorporated acute studies, if certain participants failed to fulfill the inclusion and exclusion criteria, we confined our data extraction solely to the healthy athletes who satisfied these criteria. If in the included long-term studies multiple tests were conducted at different time points before and after the intervention, we only extracted baseline data before the intervention and immediate data after the intervention. If in the included long-term studies, multiple CMDT groups were compared with a control group (CON), we selected only the CMDT group with the lowest difficulty to minimize the impact of CMDT difficulty on intervention effects.

### 2.4 Risk of bias

To minimize potential biases in our result, we rigorously controlled the quality of the included literature and conducted quality assessments independently by two researchers (Junyu Wu and Peng Qiu). For the assessment, we adopted a modified version of the Quality Index Scale (Downs and Black, 1998), which reduced the number of evaluation questions from the original 24 to 14. This modified scale has been recently utilized and widely applied in similar studies within the field of sports (Bujalance-Moreno et al., 2019). The key dimensions assessed by the scale include: (1) clarity of the objectives, (2) clarity of the description of the primary outcomes to be measured, (3) clarity of the description of participant characteristics, (4) clarity of the description of the primary results, (5) presence of random variability estimation in the primary results, (6) clarity in the reporting of specific *p*-values associated with the primary results, (7) representativeness of the selected participants, (8) implementation of blinding, (9) clarity in describing data mining if utilized for primary data, (10) accuracy of the outcome measures for the primary results, (11) appropriateness of statistical tests employed for the primary results, (12) allocation of subjects (experimental design, case–control, or cohort study), (13) random assignment of subjects to intervention groups, and (14) adjustment for confounding factors in the analysis of the main conclusions. Each question is typically answered in a “Yes/No” format, where each “Yes” response earns one point and a “No” response scores zero, thereby enabling the scoring of the overall quality of the study. The findings from the assessment of risk bias are detailed in Tables 1, 2.

**Table 1:** Literature quality assessment of acute effects studies.

Item code	1	2	3	6	7	10	12	15	16	18	20	22	23	25	Final score
Amico and Schaefer (2022)	1	1	1	1	1	1	1	U	1	1	1	1	U	1	1
Fleddermann and Zentgraf (2018)	1	1	1	1	1	1	0	U	1	1	1	1	U	1	0.92
Gabbett et al. (2011)	1	1	0	1	1	1	0	U	1	1	1	1	U	1	0.83
Gabbett and Abernethy (2012)	1	1	1	1	1	1	1	U	1	1	1	1	U	1	1
Gabbett and Abernethy (2013)	1	1	0	1	1	1	1	U	1	1	1	1	U	1	0.92
Sarto et al. (2020)	1	1	1	1	1	1	1	U	1	1	1	1	U	1	1
Schaefer and Scornaienchi (2020)	1	1	1	1	0	1	1	U	1	1	1	1	U	1	0.92
Gutiérrez-Davila et al. (2017)	1	1	1	1	1	0	0	U	1	1	0	1	U	1	0.75
Van Biesen et al. (2018)	1	1	1	1	1	1	0	U	1	1	1	1	U	1	0.92
Laurin and Finez (2020)	1	1	1	1	0	1	1	U	1	0	0	1	U	1	0.75

U, unclear. Item 1, Is the hypothesis/aim/objective of the study clearly described?; Item 2, Are the main outcomes to be measured clearly described in the Introduction or Methods sections?; Item 3, Are the characteristics of the patients included in the study clearly described?; Item 6, Are the main findings of the study clearly described?; Item 7, Does the study provide estimates of the random variability in the data for the main outcomes?; Item 10, Have actual probability values been reported (e.g., 0.035 rather than < 0.05) for the main outcomes, except where the probability value is less than 0.001?; Item 12, Were those subjects who were prepared to participate representative of the entire population from which they were recruited?; Item 15, Was an attempt made to blind those measuring the main outcomes of the intervention?; Item 16, If any of the results of the study were based on “data dredging,” was this made clear?; Item 18, Were the statistical tests used to assess the main outcomes appropriate?; Item 20, Were the main outcome measures used accurate (valid and reliable)?; Item 22, Were study subjects in different intervention groups (trials and cohort studies), or were the cases and controls (case–control studies) recruited over the same period?; Item 23, Were study subjects randomized to intervention groups?; Item 25, Was there an adequate adjustment for confounding in the analyses from which the main findings were drawn?

**Table 2:** Literature quality assessment of chronic effects studies.

Item code	1	2	3	6	7	10	12	15	16	18	20	22	23	25	Final score
Casella et al. (2022)	1	1	0	1	1	1	1	U	1	1	1	1	U	1	0.92
Gabbett et al. (2011)	1	1	0	1	1	0	1	U	1	1	1	1	U	1	0.83
Lucia et al. (2021)	1	1	0	1	1	1	1	U	1	1	1	1	U	1	0.92
Lucia et al. (2023)	1	1	0	1	1	1	1	U	1	1	1	1	U	1	0.92
Lucia et al. (2023)	1	1	0	1	1	1	1	U	1	1	1	1	U	1	0.92
Romeas et al. (2019)	1	1	0	1	1	1	1	U	1	1	1	1	U	1	0.92
Fleddermann et al. (2019)	1	1	1	1	0	1	1	U	1	1	1	1	U	1	0.92

U, unclear; Item 1, Is the hypothesis/aim/objective of the study clearly described?; Item 2, Are the main outcomes to be measured clearly described in the Introduction or Methods sections?; Item 3, Are the characteristics of the patients included in the study clearly described?; Item 6, Are the main findings of the study clearly described?; Item 7, Does the study provide estimates of the random variability in the data for the main outcomes?; Item 10, Have actual probability values been reported (e.g., 0.035 rather than <0.05) for the main outcomes, except where the probability value is less than 0.001?; Item 12, Were those subjects who were prepared to participate representative of the entire population from which they were recruited?; Item 15, Was an attempt made to blind those measuring the main outcomes of the intervention?; Item 16, If any of the results of the study were based on “data dredging,” was this made clear?; Item 18, Were the statistical tests used to assess the main outcomes appropriate?; Item 20, Were the main outcome measures used accurate (valid and reliable)?; Item 22, Were study subjects in different intervention groups (trials and cohort studies), or were the cases and controls (case–control studies) recruited over the same period?; Item 23, Were study subjects randomized to intervention groups?; Item 25, Was there an adequate adjustment for confounding in the analyses from which the main findings were drawn?

## 3 Results

**Figure 1:**
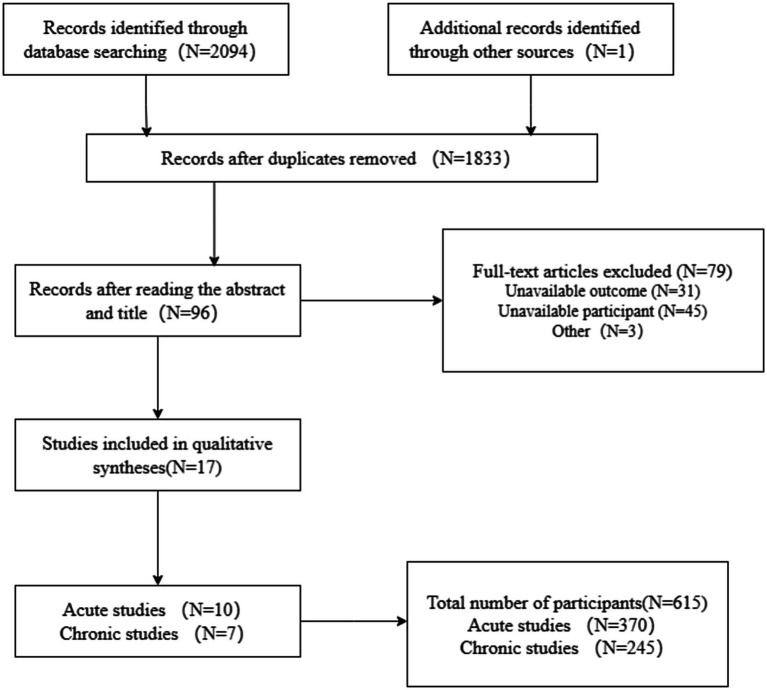
Flow chart of literature search steps.

Figure 1 illustrates the flowchart detailing the literature retrieval process. As Figure 1 indicates, our search through the aforementioned four databases yielded 2,094 articles. Duplicate entries were eliminated using Endnote 9.1X, leaving a total of 1,833 articles. An initial screening, predicated on the examination of titles and abstracts, pinpointed documents that satisfied the inclusion and exclusion criteria, leading to the selection of 96 articles that necessitated a detailed review. Ultimately, 28 studies were incorporated into the review, with 21 studies examining the acute effects of ST and DT, and 7 studies evaluating long-term effects. Two independent researchers (Junyu Wu and Peng Qiu), conducted each step of the process. In instances of disagreement, a third researcher (Youqiang Li), jointly adjudicated on the inclusion of the document.

Tables 1, 2 present the quality assessment results of the acute and chronic studies, respectively. According to Table 1, the highest quality score among the acute studies was 1, and the lowest was 0.75. According to Table 2, the highest quality score among the chronic studies was 0.92, and the lowest was 0.83. These result indicates that the articles included in our study demonstrate a moderate to high level of quality.

Table 3 shows the cognitive-motor performance of subjects during the transversal ST and CMDT evaluation in each acute study (total 10 articles). The primary objectives of these studies can be categorized as follows: (1) To simulate a match or a critical part of a match (with a much higher cognitive load) using the CMDT in order to assess athletes’ mastery of motor skills in this complex scenario. (2) Investigating the performance differences between high-level and low-level athletes in ST and CMDT situations, thereby demonstrating the superior sensitivity of CMDT acute assessments over ST. These two types of studies usually involve creating a situation highly similar to a particular sport, where athletes complete a primary sport-related task (such as tennis, volleyball, football, table tennis, soccer, fencing, etc.) while simultaneously undertaking a cognitive task (primarily auditory, visual, memory, or arithmetic tasks). Except for one sub-group in one study that reported superior DT performance under CMDT conditions compared to ST (the study of Amico and Schaefer, 2022 where high-level tennis players achieved a higher number of hits under DT conditions compared to ST), all acute studies reported superior performance under ST than DT, regardless of whether it is cognitive or motor performance.

**Table 3:** Effects of acute CMDT on athletes’ cognitive-motor performance.

References	Participant	Age	Level	Task	Major outcome	Result	Value of *p*
Amico and Schaefer (2022)	24 tennis athletes medium expertise (12) high expertise (12)	20.2 ± 2.9 21.9 ± 3.6	German Tennis Federation 1–23	ST: tennis returns.	3-back score, number of hits	3-back score ST > DT	<0.05
				DT:3-back task+ tennis returns.		Number of hits ST > DT (medium) ST < DT (High)	<0.05
Fleddermann and Zentgraf (2018)	24 beach volleyball players (21 women and 3 men)	19.2 ± 4.2	National	ST: volleyball block	Decision-making, jump height, and stride length	Jump height = ST > DT	<0.05
				DT: volleyball block +visual stimulus		Stride length = ST > DT	<0.05
						Decision-making (error) = ST < DT	Not reported
Gabbett et al. (2011)	37 ruby players	17.3 ± 0.9	National state	ST:2-on-1 situation	Draw and pass proficiency, verbal reaction times, tone recognition accuracy	Draw and pass proficiency: ST > DT (low) ST > DT (high)	low:<0.05
	20 high-level	17.1 ± 0.2		DT:2-on-1 situation + verbal tone recognition task			high: >0.05
						verbal reaction times: ST < DT	<0.05
	17 lesser-level					Tone recognition accuracy: ST > DT	<0.05
Gabbett and Abernethy (2012)	12 high-level ruby players	22.9 ± 0.9	National	ST:2-on-1 situation/3-on-2 situation	Cognitive errors, draw and pass proficiency, verbal reaction time, response accuracy	Verbal reaction time: ST < DT	<0.05
				DT:2-on-1 situation/3-on-2situation + arithmetic manipulation		Response accuracy: ST < DT	<0.05
						Draw and pass proficiency (2 on 1):ST < DT	>0.05
						Draw and pass proficiency (3 on 2):ST > DT	>0.05
Gabbett and Abernethy et al. (2013)	88 rugby league players		National	ST: anticipation test	Verbal reaction time, response accuracy	Whether primary or secondary mission	>0.05
						Verbal reaction time: ST < DT	
				DT: anticipation test+ verbal tone recognition		Response accuracy: ST < DT	>0.05
Sarto et al. (2020)	19 endurance athletes	28.32 ± 4.59	Not reported	ST: DPB/SPB	DPB performance, SPB performance, cognitive performance	DBP performance: ST > DT	END:<0.05
						SBP performance: ST > DT	TA:>0.05
	16 team athletes	23.44 ± 2.49		DT: SPB + Subtractive tasks		Cognitive performance	<0.05
Schaefer and Scornaienchi (2020)	22 table tennis players (7 women and 15 men,11 experts and 11 novices)	25.5 ± 2.6	Not reported	ST: technical and tactical task	Accuracy; working memory capacity	Technical-tactical accuracy: experts = ST > DT, novices = ST > DT; working memory capacity: experts = ST > DT, novices = ST > DT	<0.05
		23.6 ± 2.2		DT: technical and tactical task + working memory task (3-back test)			<0.05
							<0.05
Van Biesen et al. (2018)	103 athletes (33females and 70 males)	22.0 ± 2.4	Amateur	ST: multiple object tracking task	Multiple object tracking task accuracy, static balance control performance	Multiple object tracking task accuracy: ST > DT	<0.05
				DT: multiple object tracking task+ balance task		Performance in the balance task: ST > DT	<0.05
Gutiérrez-Davila et al. (2017)	25 fencing players (15 men and 10 women)	Homens:21.1 ± 4.9	Elite	ST: attacking actions against an opponent after a pre-established visual stimulus	Reaction time; speed in the attacking actions; technical-tactical offensive and defensive performance	Reaction time: DT > ST	>0.05
		Mulheres:21.4 ± 2.3		DT: an attentional task in which players were required to react differently to visual stimuli in the trunk and the head.		Speed of attack actions: ST > DT	<0.05
						Technical-tactical defensive performance: ST > DT	<0.05
Laurin and Finez (2020)	90 male soccer players	Study 1:19.2 ± 1.3	College	ST: juggling performance	Performance in juggling performance	Technical performance = ST > DT	<0.05
		Study 2:19.2 ± 1.1		DT: juggling performance + perform arithmetic subtraction operations + count down from 3 by 3 from 300 juggling performance + multiplication task			
		Study 3:19.9 ± 1.3					

ST, single-task; DT, cognitive-motor dual-task; DPB, Dynamic postural balance; SPB, Static postural balance. For acute studies, a common approach is conducting mixed-design analysis of variance (ANOVA). In this Table, we have provided a concise summary of the statistical values pertaining to the main effects of task types. For a more comprehensive set of statistical details, we recommend referring to the original text.

Table 4 presents the basic information of the long-term studies included in this review. This systematic review incorporated seven long-term studies related to the impact of ST and CMDT on the cognitive-motor performance of athletes. The purpose of all long-term studies was to improve the adaptability of athletes to CMDT, with the aim of enhancing the transfer effect of general cognitive ability or specific athletic ability, thereby improving the cognitive-motor performance of athletes. Generally speaking, all included studies reported a significant improvement in most indicators of cognitive-motor performance in athletes after CMDT training intervention, with only a few indicators showing no statistical difference in improvement compared to ST training.

**Table 4:** Effects of chronic CMDT on athletes’ cognitive-motor performance.

References	Participant	Age	Level	Task	Major outcome	Result	*p*-values
Casella et al. (2022)	24 children soccer athletes	10 ± 0.4	Not reported	ST: soccer training	TOL test	TOL (error):CON>EXP	<0.05
				DT: soccer training +voice task	WISC-IV cancelation test	WISC-IV: CON<EXP	<0.05
				regimen: 10 weeks, 2 times/week, 22 min/time as a supplement to regular training		TOL (score):CON<EXP	<0.05
Gabbett et al. (2011)	21 high-level ruby players	17.3 ± 0.9	National	ST:2-on-1 situation/3-on-2 situation	Cognitive errors, draw and pass proficiency, verbal reaction time, response accuracy	Draw and pass proficiency (ST condition)	<0.05
				DT:2-on-1 situation/3-on-2 situation +arithmetic manipulation			
				training regimen:8 weeks, sessions 3–5 involved 2-on-1 drills the final three training sessions involved simple 3-on-2 drills		ST<DT draw and pass proficiency (DT condition) ST < DT	<0.05
Lucia et al. (2021)	52 basketball athletes (females 28 males 24)	16.33 ± 1.1	Semi-elite	ST: dribbling tasks	Response times, false alarms, single change tests completion time, Multiple change tests completion time	Single change tests completion time: ST > DT	<0.05
				DT: dribbling tasks+ visual task training		Multiple change tests completion time: ST > DT	<0.05
				Regimen:5 weeks, 2 times a week, 30 min/time as individual technical training		Response times: ST > DT	<0.05
						False alarms: ST > DT	<0.05
Lucia et al. (2023)	24 young male semi-elite	16.6 ± 1.1	Semi-elite	ST: dribbling tasks	5 kinds of basketball dribbling, commission error	5 kinds of basketball dribbling performance: DT > ST	<0.05
				DT: dribbling tasks+ visual task			
	Basketball players			Training regimen:5 weeks,2 times/ week,30 min/time as individual technical training		Commission error: ST > DT	<0.05
Lucia et al. (2023)	52 young semi-elite basketball players (28 females and 24 males)	16.1 ± 1.1 16.5 ± 1.2	Semi-elite	ST: dribbling tasks	single change tasks completion time, multiple change tasks completion time. Response time, commission errors.	single change tests completion time: ST > DT	<0.05
						Multiple change tests completion time: ST > DT	<0.05
				DT: dribbling tasks+ visual task		Response times: ST > DT	<0.05
				Training regimen:5 weeks,2 times/ week, 30 min/time as individual technical training		Commission errors: ST > DT	<0.05
Romeas et al. (2019)	29 badminton players (6 women and 23 men).	22.98 ± 2.77	Amateur	ST:3D-MOT training	Visual behavior, working memory capacity, decision-making tasks accuracy, reaction times,	Reaction times: ST > DT	<0.05
				DT:3D-MOT training + badminton birdie interceptions		Decision-making tasks accuracy: ST < DT	<0.05
				Training regimen:12 times (30 min/time 9times, 90 min/time 3times)		Working memory Capability: ST < DT	<0.05
						Visual behavior: no significant	>0.05
Fleddermann et al. (2019)	43 beach volleyball players, intervention group 22 (2 men and 20 women) and control group 21 (5 men and 16 women)	Intervention group:16.38 ± 1.7	Elite	DT: the specific or nonspecific motor task of volleyball +3D-motion task. training regimen: 8 weeks with 2time/week, 30 min/time. Each block comprised 3 sessions, 8 min each with a 3 min break in-between.	Working memory capacity; jump height in a specific task (beach volleyball); accuracy in 3D motion task; attentional capacity; processing speed	Performance in the 3D motion task: DT > ST	<0.05
						Sustained attention: DT > ST	<0.05
						Processing speed: DT > ST	<0.05
		Control group:21.38 ± 4.53				Jump height: ST > DT	<0.05
						Working memory capacity: no significant difference between groups and time.	>0.05

ST, single-task; DT, cognitive-motor dual-task; 3D-MOT, three-dimensional multiple object tracking; TOL, Tower of London; 5 kinds of basketball dribbling including crossover, double crossover, between legs, crossover + between legs, between legs + behind.

The seven studies were individually focused on various sports (football, rugby, basketball, badminton, beach volleyball), and as a result, the athletic tasks were formulated to reflect the particular skills demanded by each of these sports. In six out of the seven studies, cognitive tasks involved visual response tasks or 3D multi-target tracking tasks, and only one study implemented the task through auditory stimulation. Given the different demands for CMDT by different sports, coupled with significant variances in specific athletic tasks, considerable heterogeneity exists in the design of intervention methods in different studies. The methods for measuring outcome indicators also varied. In the arrangement of training plans, differences are present in key variables such as intervention duration, frequency, among different studies (including one study that did not report the duration of single interventions and weekly training frequency). Within the six long-term studies detailing the duration of individual interventions, the length of single training sessions fluctuated between 22 and 90 min. The predominant training frequency was set at twice a week, and the intervention periods extended from 5 to 10 weeks.

## 4 Discussion

This systematic review amalgamates and analyzes relevant literature, revealing that that athletes typically experience a degradation in performance under CMDT compared to ST when assessed transversally. However, the implementation of long-term CMDT has been observed to augment cognitive-motor performance in athletes. Within the body of literature investigated in this review, acute CMDT studies are primarily employed to evaluate athletes’ tactical skill levels. Conversely, long-term CMDT is treated as a supplementary training modality designed to induce positive adaptation in athletes through sustained stimuli, thereby bolstering cognitive-motor performance in specified contexts. These findings substantiate the long-term advantages of CMDT in the domain of athletic training. Based on the existing body of evidence, CMDT emerges as a potent adjunct training tool within the sphere of sports training, poised to enhance the cognitive-motor performance in athletes engaged in cognitively demanding sports. Additionally, these insights lay the groundwork for sports training professionals, including coaches and athletes, to acquire a more nuanced comprehension of the time-related dynamics and evolutionary trends in CMDT training. This knowledge will empower them to craft or refine training regimens to optimize athletes’ performance.

Our findings are consistent with those of previous studies, which concluded that transversal CMDT evaluation typically lead to a sharp decline in athletes’ performance compared to ST (Moreira et al., 2021). However, as the athlete gradually acclimatizes to this unique stimulus, sustained exposure to CMDT ultimately leads to an improvement in their cognitive-motor performance. This abrupt reduction in performance in response to an acute CMDT can be accounted for by the cognitive load theory (Baumeister, 1984; Fuster et al., 2021). According to this theory, an individual’s working memory capacity is finite. In this context, type 2 processing refers to slow, deliberate, and effortful cognitive activities, which are more resource-intensive and can only manage a limited amount of information within a specified period (Furley et al., 2015). In a CMDT scenario, when an ancillary task abruptly elevates the cognitive load, a “choking” effect ensues, ultimately resulting in a sharp decline in performance (Baumeister, 1984; Moher et al., 2009). This performance drop appears to be closely tied to the level of the athlete’s training and the complexity of the CMDT. For example, studies have shown that athletes of higher competence deliver superior performance under CMDT conditions (Gabbett and Abernethy, 2012; Schaefer and Scornaienchi, 2020; Amico and Schaefer, 2022). Interestingly, in Amico et al.’s study, elite tennis players even hit the ball more in the DT than in the ST situation (Amico and Schaefer, 2022). According to DT effect model as described by Plummer et al. (2014), the exceptional performance observed in Amico 2022’s study under CMDT conditions may be indicative of the elite athletes’ ability to optimize task management and resource allocation, resulting in enhanced performance. Notably, Gabbett et al. (2011) even used a specialized CMDT test in rugby as a tool to assess the technical level of national-grade rugby athletes. While earlier studies suggested that athletes with a wealth of professional experience, attributed to their superior working memory capacity, can excel under CMDT conditions, recent studies indicate that a superior working memory capacity does not invariably lead to improved DT performance (Laurin and Finez, 2020). Although a majority of studies confirm the importance of working memory capacity in enhancing DT performance (Baumeister, 1984; Furley et al., 2015; Moreira et al., 2021), additional studies are necessary to unravel this intricate mechanism. Further, there is a discernible correlation between the complexity of CMDT and performance (Gabbett et al., 2011; Gabbett and Abernethy, 2012). In an assessment of this correlation, (Gabbett et al., 2011) compared the CMDT performance of national-level rugby players under 2 vs. 1, 3 vs. 2, and 4 vs. 3 passing scenarios, revealing a decline in performance as the offense-defense scenarios grew increasingly complex. Importantly, their series of studies have found that, under real match conditions, the frequency of utilizing these techniques in 2 vs. 1, 3 vs. 2, and 4 vs. 3 scenarios progressively decreases. Due to a high turnovers rate, athletes barely employ this technique in 4 vs. 3 situations.

In actual sports competitions, the influence of acute Cognitive-Motor Dual-Task (CMDT) on the cognitive-motor performance of athletes is more intricate than initially apparent. It is not only subject to interference from the surge in cognitive load under DT conditions, but the physiological load on the athletes also impacts their performance (Schapschröer et al., 2016). As athletes grow increasingly fatigued, their cognitive function correspondingly declines, leading to a rise in decision-response time and error rate (Schapschröer et al., 2016). Conversely, when the cognitive load on an athlete surges, type 2 processing allocates more working memory to the cognitive task. The scattered attention subsequently results in a significant drop in the execution efficiency of the motor task, culminating in an overall performance decline (Baumeister, 1984). Given that the cognitive-motor performance of athletes on the field is influenced by the interplay of physiological load and cognitive load, we posit that it is necessary to introduce CMDT as a supplementary training regimen in sports that demand high cognitive loads, such as team ball games. This strategy will help athletes better manage the intricacies of performing simultaneous cognitive and motor tasks during competition, potentially leading to improved performance. Furthermore, an athlete’s capability to swiftly and accurately interpret the dynamic elements of the game (Piras et al., 2014; Roca et al., 2018; Li et al., 2023), such as displacement direction and velocity of teammates, opponents, and objects like the ball, is pivotal. Rapidly adapting to these ever-changing spatial and temporal factors is a critical aspect of cognitive-motor coordination (Li et al., 2023). In team ball sports, for example, players must not only be cognizant of the present positions of others but also adept at predicting and responding to their potential trajectories and speeds. This heightened spatial–temporal awareness is essential for making strategic decisions and executing precise physical actions (Voyer and Jansen, 2017). Consequently, incorporating training elements in CMDT that emphasize skill development in perceiving and responding to these dynamic displacements is vital for optimizing cognitive and motor task performance.

While previous studies have supported the potential benefits of long-term DT training for athletes, the systematic review of Moreira et al. (2021) did not specifically discuss the application of CMDT in the field of sports training. Considering the cognitive-motor demands and the interaction between physiological and cognitive loads in athletes’ real-life competitive scenarios, we excluded all DT studies that focused on a single cognitive or motor task. The results remained consistent. However, among all the included long-term studies, only few studies quantified athletes’ cognitive load and physiological load, and none of the studies objectively quantified physiological load using specific metrics. This lack of quantification of physiological load poses challenges in explaining the long-term effects of CMDT. Subsequent research should include pertinent measures to gauge load, enhancing the understanding of the sustained effects of CMDT.

Currently, the underlying mechanisms through which CMDT enhances cognitive-motor performance in athletes remain unclear. Prior research indicates that DT training enhances the evolution of perceptual-cognitive strategies by augmenting attentional distribution and aiding in the discernment of crucial details relevant to the task (Bherer et al., 2008). For instance, Ducrocq et al. (2017) found that DT training significantly increased the duration of fixations, thereby providing more informative cues for tactical analysis and decision-making. Additionally, the Allocation and Scheduling Hypothesis (Strobach, 2020), as a classical theory explaining the long-term training effects of CMDT, offers another perspective on athletes’ performance improvements following CMDT training. This hypothesis posits that CMDT training enhances the allocation and scheduling of cognitive resources in integrated tasks, thereby enhancing CMDT performance. For example, Fleddermann and Zentgraf (2018) observed improvements in sustained attention and processing speed, contributing to enhanced CMDT performance. Furthermore, recent studies by Lucia et al. (2021, 2023), utilizing event-related potentials in a series of investigations involving semi-professional adolescent basketball players, suggest that the potential mechanisms underlying the long-term effects of CMDT may involve enhanced anticipatory brain processing capabilities in the prefrontal cortex along with increased post-perceptual activity associated with decision-making. They propose that CMDT can modulate cognitive functioning through neuroplasticity processes in the brain to achieve specific sport-related goals (Lucia et al., 2021).

Although this systematic review provides new insights into the application of CMDT in sports training, it has several limitations. Firstly, the systematic review included studies that generally lacked detailed descriptions of key training variables. For instance, intensity, inter-set rest periods, cognitive load, and physiological load were often inadequately reported. Even when some studies described athletes’ physiological and cognitive loads, the measurement methods were often subjective, lacking objective indicators. This makes it difficult to discern the relationship between load and adaptation while also reducing the practical applicability of research findings in real-world settings. Secondly, the majority of studies, especially those investigating acute effects, tended to be conducted in laboratory settings, which presents a challenge in simulating game elements as closely as possible. To promote the widespread adoption of CMDT training methods in sports, future studies should aim to conduct studies in sports-specific environments. Previous studies have suggested that conducting small-sided games or game simulations on sports fields helps replicate real tactical and technical situations (Davids et al., 2013), which would be more meaningful in the context of sports training. Lastly, the notable variation in participant characteristics, such as age, gender, and sports proficiency, combined with diverse methodological approaches in the field, has presented challenges in synthesizing research findings. To address this issue, future studies should focus on minimizing these differences by adopting more uniform and standardized methods in Cognitive-Motor Dual-Task (CMDT) training research.

In summary, future CMDT experimental research aiming to enhance athletes’ cognitive-motor performance should be conducted as much as possible in real sports settings, with an emphasis on detailed reporting of key training variables to better facilitate optimal cognitive-motor performance in athletes.

## 5 Conclusion

This systematic review posits that athletes generally exhibit a decline in cognitive-motor performance when assessed transversally CMDT, as compared to ST. However, in contrast to ST training, athletes demonstrate a more pronounced improvement in cognitive-motor performance following prolonged CMDT training. Our study provides new insights into the application of CMDT in the field of sports training. Practitioners can utilize CMDT to assess athletic skill levels or optimize cognitive-motor performance of athletes, taking into account the specific needs of each sport.

## Data availability statement

The original contributions presented in the study are included in the article/Supplementary material, further inquiries can be directed to the corresponding author.

## Author contributions

JW: Writing – original draft, Writing – review & editing, Conceptualization, Data curation, Supervision. PQ: Data curation, Formal analysis, Methodology, Writing – original draft. SL: Conceptualization, Data curation, Formal analysis, Writing – review & editing. MC: Data curation, Methodology, Writing – review & editing. YL: Supervision, Writing – review & editing.
