# Supplementary material for: The effects of cognitive-motor dual-task training on athletes’ cognition and motor performance
Source: Front Psychol. 2024 Feb 8;15:1284787. doi: 10.3389/fpsyg.2024.1284787 (PMC10881661; doi:10.3389/fpsyg.2024.1284787)
Supplement: Supplementary file 1 [file Table_1.docx]

Supplemental Figure


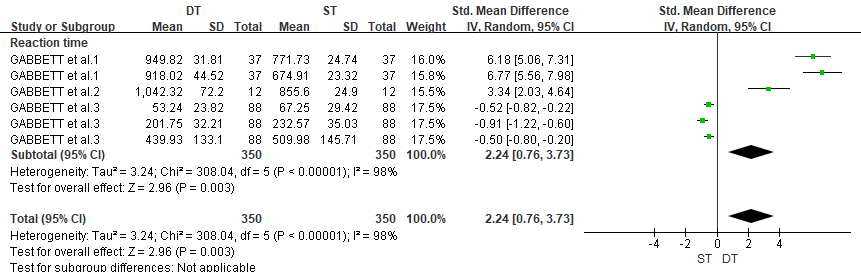


Supplemental Figure S1 Forest plot of the effect of acute CMDT on reaction time


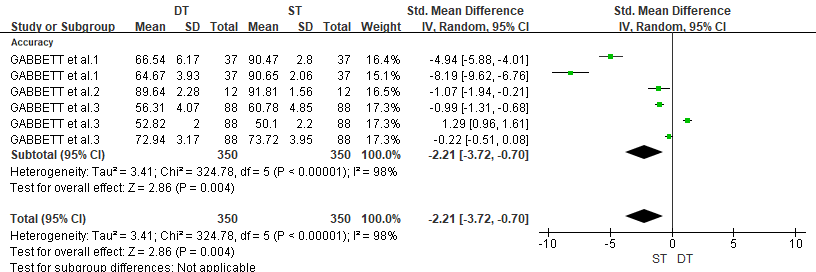


Supplemental Figure S2 Forest plot of the effect of acute CMDT on accuracy


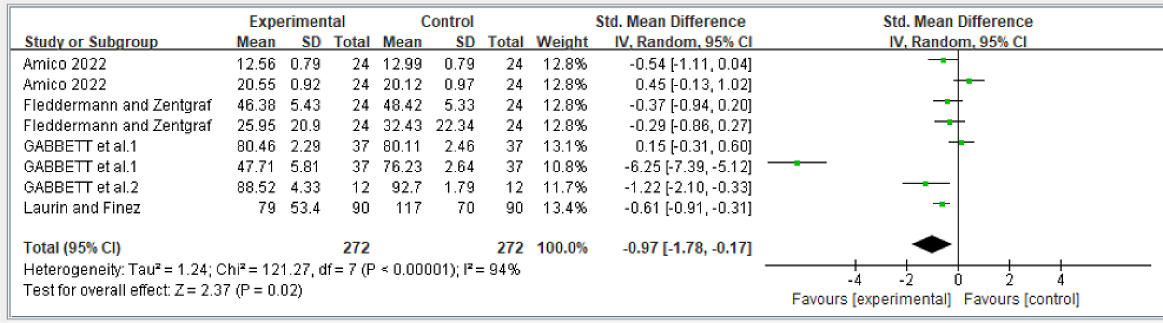


Supplemental Figure S3 Forest plot of the effect of acute CMDT on motor performance


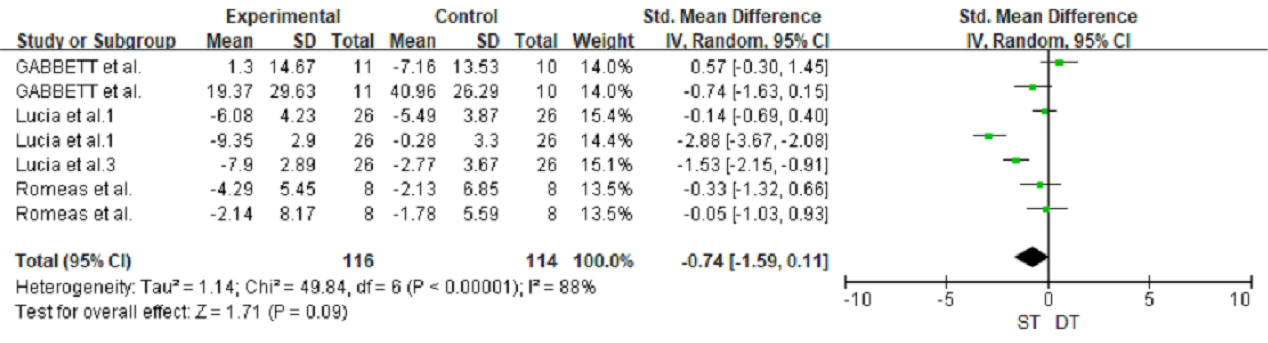


Supplemental Figure S4 Forest plot of the effect of acute CMDT on reaction time


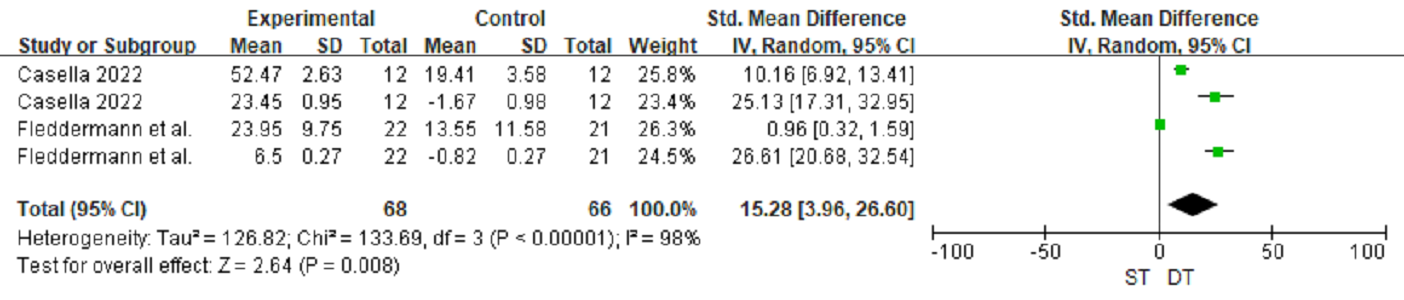


Supplemental Figure S5 Forest plot of the effect of acute CMDT on cognitive performance


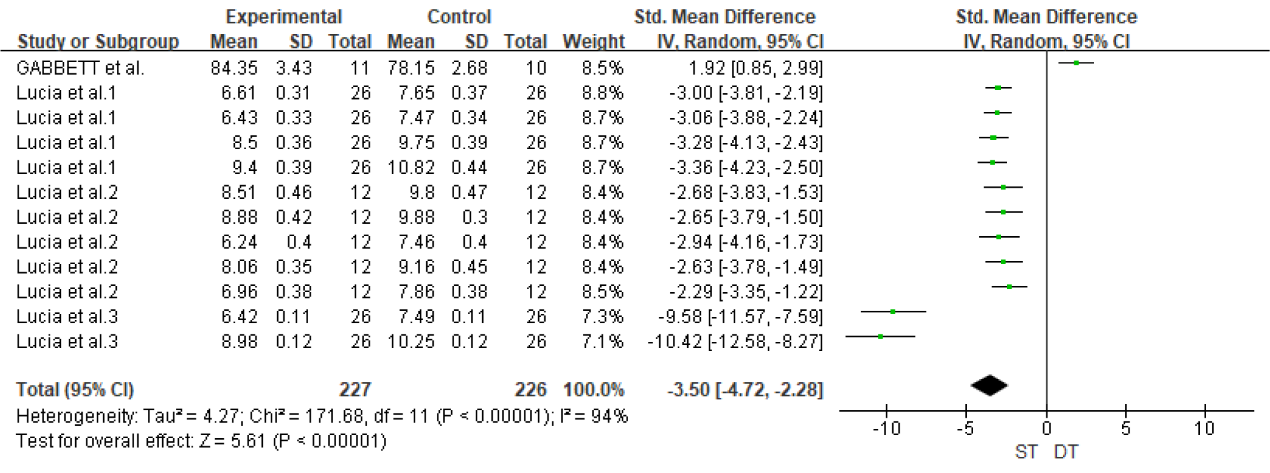


Supplemental Figure S6 Forest plot of the effect of acute CMDT on motor performance
